# Evidence of familial resemblance and family-based heritability of food intakes derived from a longitudinal cohort study

**DOI:** 10.1038/s41598-023-38326-z

**Published:** 2023-07-24

**Authors:** Mahdi Akbarzadeh, Farshad Teymoori, Parisa Riahi, Hossein Farhadnejad, Hamid Ahmadirad, Asiyeh Sadat Zahedi, Firoozeh Hosseini-Esfahani, Maryam Zarkesh, Mohammadreza Vafa, Parvin Mirmiran, Maryam S. Daneshpour

**Affiliations:** 1 Cellular, and Molecular Endocrine Research Center, Research Institute for Endocrine Sciences, Shahid Beheshti University of Medical Sciences, Tehran, 19195-4763 Iran; 2 Nutrition and Endocrine Research Center, Research Institute for Endocrine Sciences, Shahid Beheshti University of Medical Sciences, Tehran, 19395-4741 Iran; 3 Department of Nutrition, School of Public Health, Iran University of Medical Sciences, Tehran, Iran

**Keywords:** Computational biology and bioinformatics, Genetics

## Abstract

We sought to investigate the familial aggregation and family-based heritability of dietary intakes among adults in a population-based longitudinal study of the Tehran Lipid and Glucose Study (TLSG). Total of 4359 males and 5439 females entered our study. We categorized foods into main groups based on the literature on main food groups and their subgroups among the Iranian dietary habits and food culture as follows: grains, fruits, vegetables, dairy, meats, legume, nuts, beverages, snacks, and fats. The intraclass correlation coefficients (ICC) are estimated to verify familial resemblance of dietary habits for all relative pairs and spouses. Family-based heritability is obtained using a mixed effect framework with likelihood-based approach. For almost all food groups, the correlation between parents and offsprings tended to be larger than those of siblings. Family-based heritability of food groups varies from the lowest 6.36% for snacks to the highest 25.67% for fruits, and 25.66% for legume. Our findings indicated weak-to-moderate similarities between parents' and offspring's food intakes; however, the similarity in parent–child food intakes was different, and the correlation in mother-daughter food intakes was stronger than other parent–child correlations, and almost all of dietary components showed strong family-based heritability.

The family environment directly affects the various behaviors of children and adolescents such as dietary habits and tendencies toward different foods^1^. Some researchers believed that many dietary behaviors are shaped within the family environment during the first two decades of life and these dietary behaviors track into adulthood^2–4^.

The family food availability, food types, food preparation methods and cooking style, frequency of family meals, eating in or out of the home, and some other dietary behaviors are controlled by parents^2,5^. For instance, it is indicated that the household availability of fruits, vegetables, and dairy foods is an important predictor of children’s consumption of these foods^6,7^. Besides, the genetic factors as an effective agent in food preferences help familial aggregation in dietary intakes^8^. A study among UK Female Twin Cohort indicated that genetic factors have an important influence in determining food choice and dietary habits, as a strong heritability was identified for garlic (46%), coffee (41%), fruit and vegetable sources (49%), and red meat (39%)^9^. Another Twin Study in China assessed the heritability of children's dietary intakes and showed that estimated heritabilities ranged from 1.1% (Western fast foods) to 65.8% (soft drinks) in boys and from 12.6% (eggs) to 94.6% (Western fast foods) in girls^10^.

The dietary intake resemblance between the family members was investigated in some previous studies and showed controversial findings^1,3,8,11–22^. Although some studies observed a weak-to-moderate positive correlation between parents and their children^1,3,8,11,16–20,22^, some reported no association^12–15,21^. Also, there are conflicting results regarding the question that which food groups and family members had a higher resemblance within a family. It seems that the familial aggregation for food intake is different in developed and developing countries, and is becoming weaker in modern societies^16,23^.

Most previous studies on the resemblance of parent–child dietary intakes were conducted in the United States and included children and adolescents lower than 18 years. Besides the family environment, the dietary food intake of adolescents is influenced by their peers and some countries have a meal at school^24^. Studies on the resemblance of familial dietary intakes in adulthood are rare. Lahmann et al. assessed the mother–adult offspring (higher than 18 years) resemblance in dietary intake using a community-based cohort study in Australia and they observed that the mother–offspring correlations were weak (r = 0.12–0.29) for most dietary factors and tended to be slightly higher in mother-daughter than in mother-son^16^. A previous study among the Tehran Lipid and Glucose Study (TLGS) population assessed the resemblance of dietary intakes in three generations of parent–offspring pairs^11,25^, however, the resemblance among spouses and siblings pairs has not been assessed among the Iranian population and generally, there are few studies on the food intake correlation between spouses^3,18,19^ or siblings^3,26,27^.

To our knowledge, family-based heritability of food intake was previously assessed in a few studies specially in twins^9,10,28^, and are rarely available in cohort studies^29^. We aimed to examine the familial aggregation and family-based heritability of dietary intakes among people age ≥ 18 years using the prospective population-based study of the TLGS.

## Results

### Demographic characteristics of participants

Baseline characteristics of participants and pedigree-based descriptions are provided. Totally, 9798 participants from TCGS (44.5% males) with a mean ± SD of age of 42.0 ± 15.2 years and BMI of 27.3 ± 5.0 kg/m^2^ were included in our study.

Pedigree and relative pairs information are provided in Table 1. There were 4338 families with a mean of 3.20 ± 2.89, including 3 to 32 members (2567 constituent pedigrees and 1572 singletons), 3627 sibships (1.60 ± 0.85; min = 1, max = 6). There were 15,203 first-degree relative pairs (11,576 Parents/offspring and 3627 siblings) and 4914 s-degree relative pairs, including grandparents, avuncular, half-siblings, and cousins. Demographic characteristics of participants in terms of their age, BMI, education status, occupation status, marital status, and food intake are presented in Table. 2.

**Table 1:** Relative-pairs information of the participants.

Pairs type	Number of pairs
First-degree relative	
Parents/offspring	11,576
Siblings (3627 pairs)	
Sister–sister	866
Brother–brother	735
Brother–sister	1438
Second-degree relative	
Grandparents	3552
Avuncular	937
Half sibling	65
Cousin	360

**Table 2:** Demographic characteristics and dietary intake of study population of Tehran Lipid and Glucose Study*.

	Females = 5439	Males = 4359	P-Value
Age(year)	41.34 ± 14.9	42.83 ± 15.71	< 0.001
BMI (kg/m^2^)	27.75 ± 5.43	26.72 ± 4.37	< 0.001
Academic education (graduated), %	24.7%	30.7%	< 0.001
Occupation status (employed), %	82.8%	73.5%	< 0.001
Marital status (married), %	70.5%	74.1%	< 0.001
Dietary food groups			
Pickles (serving/1000 kcal/d)	0.07 ± 0.12	0.05 ± 0.09	< 0.001
Garlic Onion (serving/1000 kcal/d)	0.1 ± 0.13	0.08 ± 0.08	< 0.001
Total vegetables (serving/1000 kcal/d)	1.86 ± 1.06	1.42 ± 0.77	< 0.001
Sweetened dairy (serving/1000 kcal/d)	0.05 ± 0.09	0.05 ± 0.08	> 0.05
High fat dairy (serving/1000 kcal/d)	0.65 ± 0.42	0.58 ± 0.38	< 0.0001
Low fat dairy (serving/1000 kcal/d)	0.45 ± 0.41	0.38 ± 0.34	< 0.001
Doogh (serving/1000 kcal/d)	0.04 ± 0.08	0.05 ± 0.08	< 0.001
Total dairy (serving/1000 kcal/d)	1.2 ± 0.57	1.07 ± 0.51	< 0.001
Tea (serving/1000 kcal/d)	1.08 ± 0.99	1.16 ± 1.03	< 0.001
Coffee (serving/1000 kcal/d)	0.02 ± 0.07	0.03 ± 0.12	< 0.001
Artificial beverages (serving/1000 kcal/d)	0.14 ± 0.24	0.21 ± 0.3	< 0.001
Red meat (serving/1000 kcal/d)	0.25 ± 0.26	0.23 ± 0.22	< 0.001
Processed meat (serving/1000 kcal/d)	0.08 ± 0.11	0.1 ± 0.14	< 0.001
Organ meat (serving/1000 kcal/d)	0.03 ± 0.08	0.05 ± 0.14	< 0.001
White meat (serving/1000 kcal/d)	0.93 ± 0.62	0.97 ± 0.65	0.001
Total meat (serving/1000 kcal/d)	1.09 ± 0.62	1.14 ± 0.64	0.001
Pizza (serving/1000 kcal/d)	0.03 ± 0.07	0.04 ± 0.07	< 0.001
Snacks (serving/1000 kcal/d)	0.9 ± 0.63	1.01 ± 0.68	< 0.001
Salty snacks (serving/1000 kcal/d)	0.11 ± 0.18	0.1 ± 0.16	0.004
Nuts (serving/1000 kcal/d)	0.12 ± 0.18	0.12 ± 0.18	> 0.05
Refined grain (serving/1000 kcal/d)	1.1 ± 1.12	1.34 ± 1.29	< 0.001
Whole grain (serving/1000 kcal/d)	1.62 ± 1.39	1.99 ± 1.49	< 0.001
Legume (serving/1000 kcal/d)	0.16 ± 0.16	0.16 ± 0.15	> 0.05
Egg (serving/1000 kcal/d)	0.13 ± 0.12	0.14 ± 0.14	0.0001
Salt (serving/1000 kcal/d)	0.92 ± 1.63	0.68 ± 1.03	< 0.001
Dry Fruits (serving/1000 kcal/d)	0.02 ± 0.07	0.02 ± 0.07	> 0.05
Fruits (serving/1000 kcal/d)	1.57 ± 1.18	1.33 ± 1.03	< 0.001
Fruits Juices (serving/1000 kcal/d)	0.09 ± 0.18	0.09 ± 0.17	> 0.05
Butter (serving/1000 kcal/d)	0.38 ± 0.67	0.42 ± 0.65	0.003
Mayonnaise (serving/1000 kcal/d)	0.18 ± 0.26	0.17 ± 0.24	0.050

*Data are presented as mean ± standard deviation for continuous variables and percent for categorical variables.

### Familial aggregation

**Figure 1:**
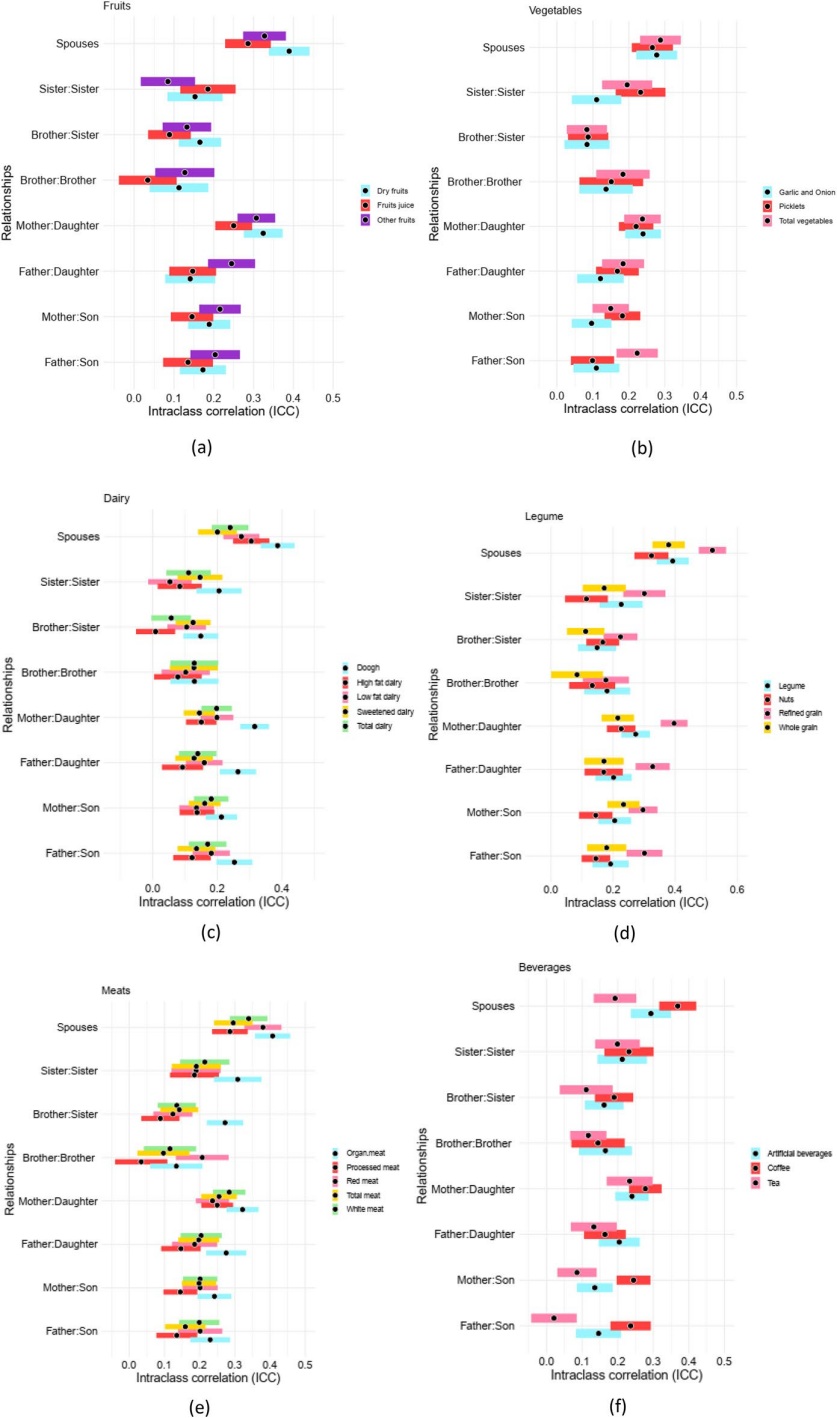

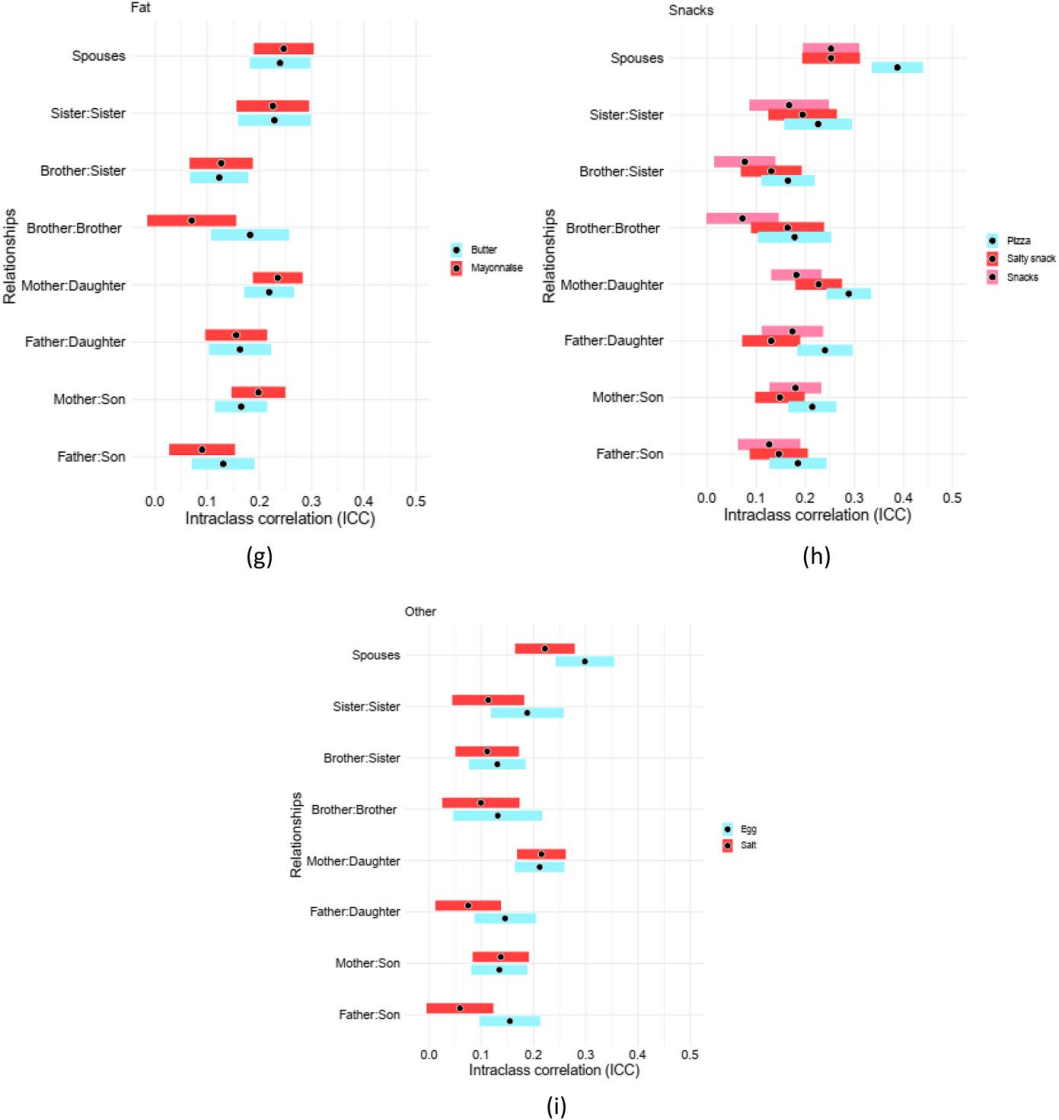
Pairwise interclass correlations (ICC) for all food groups for all first-degree dyads. Figures provide the pairwise intraclass correlation (ICC) for all types of dyads within first-degree relationship (Sibling-Sibling, Parent-Offspring, and Spouses). Accordingly, (**a**) presents ICCs for fruit derivatives, (**b**) for garlic/onion, pickles, and vegetables, (**c**) for each kind of diary intake, (**d**) for nuts, legume, and grain, (**e**) all kinds of meat (red, white, processed, etc.), (**f**) for beverages, tea, and coffee, (**g**) for butter and mayonnaise, (**h**) for pizza and snacks, and (**i**) for eggs and salt consumption along with a 95% confidence interval (CI).

All types of first-degree relatives are considered for their pairwise interclass correlations (ICC) for all food groups. Figure 1a–i and Supplementary File 1, S1 Table 1–9, provide the ICCs for all of food groups. For almost all food groups, the correlation between parents and offsprings inclined to be larger than those of siblings.

Considering meats category, including red, white, processed, organ, and total meat, the largest ICC belongs to organ meat with ICC_mother:daughter_:32.19% (se:2.31%).For dairy intake, the highest ICC belongs to doogh, with ICC_mother:daughter_ 31.56% (se:2.27%), and the smallest correlation belongs to high fat dairy for brother:sister with ICC:0.88% (se:3%).

Alternatively, trend changes for fat intake for some pairs, as the mayonnaise consumption has the largest correlation between mothers and daughters, and in the next level belongs to sister:sister pairs, with ICCs 23.50% (SE:2.43%) and 22.54% (SE:3.55%), respectively.

Coming to the fruit consumption, ICC_mother:daughter_ for dry fruits, is estimated to be 32.42% (SE:2.49%), and ICC_brother:brother_ is 3.42% (SE:3.7%) for fruit juice, respectively, as the largest and smallest pairwise correlation between family members. Pairwise vegetable, pickle, onion, and garlic intake correlation had the largest amount for mother:daughter group, with 30.70% (SE:2.54%) for garlic and onion. Considering legume and grains, the largest correlation belongs to refined grain with ICC_mother:daughter_:39.66% (SE: 2.2%).

When it comes to pizza and snack consumption, we provide that mothers and daughters, and in the second level, fathers and daughters have the highest correlation, as ICCs for eating pizza are 28.86% (SE:2.31%), and 24.01% (SE:2.28%), respectively. For snacks, mother:daughter correlation is the largest among others, with ICC being 18.19% (SE:2.62%). Considering the egg and salt consumption, findings show that the mothers and daughters are of highest ICC among other pair, with their ICCs both equal to ~ 21%.

Coming to the spouses, we provide that pairwise correlations between spouses are significantly higher than those of other pair groups for all food categories. As, the highest ICC belongs to refined grains with ICC: 52.01% (SE:2.24%), and lowest ICC belongs to tea consumption with ICC: 19.24% (SE:3%).

### Family-based heritability

Family-based heritability of food groups varies from the lowest 6.36% (SE:1.18%) for snacks to the highest 25.67% (SE:3.27%) for fruits, and 25.66% (SE:3.27%) for legume. Table 3 provides the family-based heritability of food groups of all categories.

**Table 3:** Family-based heritability of food intake.

Food groups	h^2^ (%)	Standard Deviation
Dairy		
Sweetened dairy	7.60	0.0373
High fat dairy	12.83	0.0361
Total dairy	12.96	0.036
Low fat dairy	11.94	0.0363
Doogh	22.93	0.0335
Vegetables		
Total vegetables	8.63	0.037
Garlic and Onion	12.40	0.0362
Pickle	20.98	0.034
Beverages		
Artificial beverages	10.13	0.0367
Tea	14.00	0.0358
Coffee	23.45	0.0321
Fruits		
Fruits Juices	12.05	0.0363
Dry fruits	18.12	0.0347
Fruits	25.67	0.0327
Fat		
Butter	12.39	0.0362
Mayonnaise	19.27	0.0345
Snacks		
Salty snacks	14.00	0.0358
Snacks	6.36	0.0118
Pizza	20.57	0.0341
Other		
Egg	15.49	0.0354
Salt	15.57	0.0354
Legume and grains		
Nuts	16.78	0.0351
Whole grain	17.23	0.035
Refined grain	22.52	0.0336
Legume	25.66	0.0327
Meats		
Organ meat	17.51	0.0349
White meat	20.73	0.0341
Red meat	24.39	0.0331
Processed meat	14.49	0.0357
Total meat	19.37	0.0344

## Discussion

In the current study, based on nationally representative data collected in Iran, we have assessed the possible familial resemblance in various dietary intakes, including food groups, food items, nutrients, and drinks, particularly between parents and their children (aged ≥ 18 years old). It is also the first study examining the Iranian population's family-based heritability of food groups. The results of the current study suggest weak-to-moderate similarities between the food intakes of spouses and also food intakes of parents and offspring. Generally, the possible familial resemblance in food intake can be influenced by heritability factors, environmental, cultural, and social conditions between family members living in the same household, and social homogamy between spouses. In this study, our important and exciting results showed an insight into the extent of parental effects on offspring’s dietary intakes or food habits and revealed a moderate to strong resemblance, in terms of correlations, in several food consumptions within families (especially remarkable correlation between spouses and mother: daughter). Also, based on our findings, the degree of correlation between spouses and parents-children and siblings regarding various food groups can be wide (ICC: less than 0.1 to more than 0.5). Our findings suggested that spouses correlations were stronger than parent–offspring correlations or siblings correlations for the various healthy or unhealthy food intakes, including fruits (dry fruits, fruit juices, and other fruits), vegetables (total vegetables, pickles, garlic and onion), dairy (low-fat dairy, high-fat dairy, sweetened dairy, doogh, and total dairy), meats (red meats, processed meats, organ meats, white meats, and total meats), some beverages (artificial beverages and coffee), fats (butter and Mayonnaise), snacks, and other foods (egg and salts).

Our findings are comparable with the results of previous studies that assessed the mother–father or parent–child dietary intake correlations^11,16–19^. Our results are in agreement with the results of Robinson et al. study that showed possible moderate-to-strong dietary correlations in generally healthy spouses. Also, they reported that there are slightly greater similarities in mother–offspring dietary intakes in comparison to father–children intakes^18^. Also, investigations on the Tehranian population revealed a weak-moderate correlation between nutrient intakes of parent–offspring dyads that lived with their parents. However, this correlation for the nutrient intakes of younger and older generations with parents was very weak and non-significant^11,17^. Also, the study on TLGS population also showed that the correlation between food consumption and dietary habit in mother–offspring dyads was stronger than in father-offspring dyads^11^. Furthermore, Shrivastava et al. have observed a moderate positive correlation between dietary intake in parents-child. They revealed that dietary resemblances in mother–offspring are greater than dietary resemblances of father–offspring at both prenatal and postnatal time points^19^. A community-based cohort study in Australia provided evidence that there is only weak mother–adult offspring dietary resemblance, which was different by food groups, nutrients, and the offspring’s sex and living arrangements, also, the correlations in mother-daughter dyads were slightly stronger than in mother-son dyads for selected dietary intakes^16^.

Based on the results of the current study, parents were modestly correlated (ICC: 0.2 to 0.5) for most of the healthy foods (fruits, vegetables, nuts, legumes, whole grain, and dairy products) and unhealthy foods (salt, fast foods, sweetened beverages, and red and processed meat), suggesting a shared household effect. Also, assortative mating may play an important role in spouses’ resemblance. Furthermore, a similar and close spousal correlation regarding healthy and unhealthy foods indicates that the home environment affects the consumption of healthy and unhealthy food items to an equal extent in adults^3^. Similar to our study, the results of most previous studies support the hypothesis that the correlation in dietary intakes of parent–offspring pairs is weaker than that of spouse pairs. The weak to moderate parental–children correlations for dietary intake showed that young individuals’ food intakes could be affected by the various environmental and societal factors in addition to household factors and parental dietary habits, such as environmental factors related to society, friends and peers influence, various media, advertising, and also as well as personal factors such as self-image and self-esteem^2^. This weaker correlation coefficient could be more evident in food intake, especially in offspring who are adults and live apart from their parents after getting married^8,16^.

In the current study, we observed a stronger resemblance between the mothers of daughters in comparison to sons. Some reasons can explain this difference in results; the various biological, physiological, psychosocial, and behavioral factors in boys and girls could be responsible for these differences. Since the stages of physical growth, maturity, and the period of body development of individuals occur in different age periods based on gender and biological differences, therefore, the nutritional needs of boys and girls may differ under the influence of these factors in the age period and cause differences in nutritional choices between them during the adult life period. For example, at a certain age, boys are in early youth regarding physical growth and development, but girls are fully matured. Physiological differences in boys and girls can also play a role in the difference in parents- offspring food correlations; due to the difference in body parts and physical strength, boys may be more physically active than girls. Furthermore, the effects of the environment, parents, friends, and peers on nutritional intake and food habits can be different between boys and girls^20^. These all may help explain the differences between the resemblance of mother-daughter and other parent-offspring.

In the current study, we determined the possible family-based heritability of food groups, which indicates a weak to moderate heritability (h^2^: 6.36% for snacks and 25.67% for fruits). Among individual food items, a remarkably family-based heritable component was shown for fruits (25.67%), legumes (25.66%), red meat (24.39%), coffee (23.45%), doogh (22.93%), refined grain (22.52%), pickle (20.98%), and pizza (20.57%). According to the traditional food pattern of Iranians in families, the consumption of some food items such as red meat, refined grains, and legumes is done simultaneously and in one main meal, because the food prepared in families is such that these common food items and all family members who live in the same home have close consumption of these food items. Therefore, the family-based heritability for these food items has been high. Also, in Iranian families, most of the daily fruit consumption is usually done after dinner and simultaneously in the presence of all family members, which has caused a high heritability for fruit consumption to be observed in this study. In Iranian society, pickles and doogh are eaten as a "side dish" or “condiment” along with the main meals, which are usually shared by family members, therefore, high family-based heritability has been observed for these food items. Also, the consumption of special food items such as pizza or coffee, which are considered luxury food choices in most of the Iranian families is done at certain times when usually all family members are together, so the amount of consumption of these food items among family members is similar and close to each other. Therefore, a high heritability rate has been reported for these cases.

This study has several strengths. This is the first study that has assessed the possible dietary intake resemblance in parent pairs, parent-offspring, and siblings (aged ≥ 18 years old). Also, for the first time, family-based heritability for different individual food components was performed in the population aged ≥ 18 years old. We considered the energy-adjusted food intakes of parents-offspring better to compare the usual nutrient intakes of parents and their offspring. Also, in the current study, we focused on the separately parent and offspring food intakes by sex, which provided different perceptions of familial resemblance and family-based heritability. Furthermore, dietary information of parents-offspring pairs was obtained using a valid and reliable FFQ, which minimized the possible measurement error. Some limitations of the current study should be mentioned; we did not check the similarities for nutrient intakes of parent-young offspring dyads that lived with their parents and offspring not living at home with their parents. Also, there is no information on the number of shared meals of parent–offspring dyads that should be controlled. Furthermore, we had no data on the number of meals at restaurants or outside the home.

In conclusion, the results of this population-based study suggest a significantly greater similarity in spouses' dietary intakes compared to parent–offspring dietary intakes. This stronger similarity was observed in both healthy and unhealthy food choices. Also, the results of the current study suggest weak-to-moderate similarities between the food intakes of parents and offspring, the similarity in parent–child food intakes was different, and the correlation in mother-daughter food intakes (for both healthy and unhealthy foods) was stronger than other parent–child correlations. The lowest correlation in food intake is also seen among siblings. Furthermore, a remarkable family-based heritability was observed for several food components, including fruits, legumes, red meat, coffee, doogh, refined grain, pickle, and pizza. Further investigations with longitudinal design and high sample size are recommended to assess the family-based heritability and the intergenerational dietary effects in other populations.

## Methods and materials

### Study participants and genealogy data

The present study was conducted within the framework of the Tehran Lipid and Glucose Study (TLGS), an ongoing community-based prospective study that was conducted on a sample of residents from District No. 13 of Tehran, the capital city of Iran. The first phase of the TLGS began in March 1999, and data collection, at 3-year intervals, is ongoing; the baseline survey was a cross-sectional study conducted from 1999 to 2001, and surveys 2 (2002–2005), 3 (2006–2008), 4 (2009–2011), 5 (2012–2014), and 6 (2015–2018) were prospective follow-up surveys. Details of the TLGS design and its preliminary results have been described elsewhere^30^.

Also within the framework of TLGS, the Tehran cardio-metabolic genetic study (TCGS) as a prospective family-based cohort study with a 3-year interval follow-up period was designed to create a comprehensive genome-wide database and familial data of the Tehranian population^31^.

The TLGS participants were assessed for familial data, dietary intakes, anthropometric parameters, biochemical factors, etc. For the present study, among the participants with complete dietary data for the third (n = 3568), fourth (n = 7956), fifth (7052), and sixth (n = 7720) survey of the TLGS, in each survey those with ≥ 18 years who were not a pregnant and lactating woman, haven’t the history of cancer, myocardial infarction, cardiovascular accident, and their nutritional data were measured for the first time were selected. From the total of 10,468 included participants (third survey (n = 2944, 28.12%), fourth survey (n = 4928, 47.07%), fifth survey (1605, 15.33%), and sixth survey (n = 991, 9.46%)), 668 subjects that have under or over report of energy intake (lower than 800 and higher than 4200 kcal/d) were excluded and 9798 participants remained for the data analyses. As mentioned above respectively 75.19% and 91.54% of participants' dietary data were collected in 3- and 6-years periods.

Among these 9798 participants, 4338 families including spouses, first-degree relative pairs (11,576 Parents/offspring and 3039 siblings), and second-degree relative pairs (including 4914grandparents, avuncular, half-siblings, and cousins) were identified.

### Ethics declaration and consent form

This research was authorized by the local research ethics committee of the Endocrine Research Institute at the University of Shahid Beheshti Medical Science (Research Approval Code 96046 and Research Ethical Code: IR.SBMU.ENDOCRINE.REC.1398.135). For their involvement in the survey, all subjects provided written informed consent. This study was carried out in accordance with the Helsinki Declaration.

### Phenotype definition

#### Dietary assessments

We use a valid and reliable semi-quantitative 168-item food frequency questionnaire (FFQ) to assess dietary intakes. The reproducibility and validity of the FFQ have been assessed previously^32^.

The consumption frequency of each food item in each survey was collected during the previous year, as daily, weekly, or monthly, in a face-to-face interview by trained and skilled dieticians. Portion sizes of consumed foods were reported in household measures and then transformed into grams scale. The energy contents of consumed foods were calculated using the United States Department of Agriculture (USDA) food composition table (FCT)(available on https://fdc.nal.usda.gov/fdc-app.html). Furthermore, the Iranian FCT was used to analysis of the local food items which were not available in USDA FCT.

#### Food groups

We used some food groups based on the literature on main food groups and their subgroups among the Iranian dietary habits and food culture as follows: grains (refined grain and whole grain), fruits (including dry fruits, fruit juices, and other fruits), vegetables (total vegetables, garlic and onion, and picklets), dairy (including total dairy, low-fat dairy, high-fat dairy, sweetened dairy, and doogh), meats (including total meat, white meat, red meat, processed meat, organ meat, and egg), legume, nuts, beverages (including artificial beverages, coffee, and tea), snacks (including sweet snacks, salty snack, and pizza), and fats (including butter and mayonnaise). The intakes of these food groups and their subgroups were calculated as daily servings per 1000 kcal energy intake.

### Demographic and anthropometric assessment

Using a pretested questionnaire, an experienced interviewer collected data on age, sex, education status, occupation status, marital status, and other variables. The participant's weight, height, and waist circumference were measured using a digital scale, stadiometer, and tape meter, respectively. Body mass index was calculated as weight (kg) divided by height in square meters (m^2^).

### Statistical analysis

Food groups for each individual were adjusted by their age and were normalized for all analyses. Pedigree information was obtained using the S.A.G.E. software.

Familial Aggregation and Spousal Resemblance.

FCOR command of the S.A.G.E. software was used to estimate the intraclass correlation (ICC) coefficients of all relative pairs^20^ to verify family resemblance of dietary habits for all relative pairs and spouses. Only significant ICCs are reported (*p* < 0.05).

#### Family-based heritability

Classical likelihood-based and Bayesian approaches were used to assess the family-based heritability of food groups. Using the former, it is possible to estimate polygenic heritability and additional family correlation parameters, which perform likelihood ratio tests and generate maximum likelihood estimates assuming multivariate normality following either George–Elstone or Box-Cox transformation^32^. In the latter, a kinship matrix was used to estimate heritability. For each individual, there were fixed factors, including age and sex. A Gaussian random-effects model with a covariance structure was used. We included a random effect, k = N(0, K $G{\sigma }_{g}^{2})$) where K is a kinship matrix, and $G{\sigma }_{g}^{2}$ is the genetic variance. The response vector $y={\{y}_{i}\}$ was defined as the food groups’ level for the ith individual. Non-Gaussian outcomes were accommodated using the probit link under a Bayesian Markov chain Monte Carlo (MCMC) setting. The probit link was implemented as $\mathrm{P}\left({y}_{i}=1|{G}_{i}\right)=\Phi \left({\eta }_{i}\right)$ where Φ is the cumulative distribution function (CDF) and ${\eta }_{i}$ is a linear predictor given by:

$$\eta_{i} = \mu + \mathop \sum \limits_{k = 1}^{q} x_{ik} \beta_{ik} + g_{i} .$$

where $\mu$ is an intercept, ${x}_{ik}$ is the kth fixed factors, ${\beta }_{ik}$ is the effects associated with the kth fixed factors, and ${g}_{i}$ is a total genetic effect of the ith individual. Our Bayesian analysis was implemented using BGLR R package^33^. The number of iterations of the Gibbs sampler was 400,000, where the first 200,000 samples were discarded as burn-in. A thinning interval of 40 was used. Thus, 5000 posterior samples were used to compare the features of the posterior distribution. The convergence was visualized through trace plots of all the unknown values and computation of the Gelman-Rubin statistic for convergence below 1.03^34^.

## Supplementary Information

Supplementary Tables.

## Data Availability

The datasets generated and/or analysed during the current study are not publicly available due to containing information that could compromise the privacy of research participants but are available from the corresponding author on reasonable request.
